# Oligomannuronate prevents mitochondrial dysfunction induced by IAPP in RINm5F islet cells by inhibition of JNK activation and cell apoptosis

**DOI:** 10.1186/s13020-020-00310-4

**Published:** 2020-03-24

**Authors:** Xi Liu, Qiong Li, Xiaolei Cheng, Zhichun Liu, Xiaoliang Zhao, Shuai Zhang, Guangli Yu, Xia Zhao, Jiejie Hao

**Affiliations:** 1 Laboratory for Marine Drugs and Bioproducts, Qingdao National Laboratory for Marine Science and Technology, Qingdao, 266237 China; 2 School of Medicine and Pharmacy, Ocean University of China, Qingdao, 266003 China; 3 The Affiliated Central Hospital of Qindao University, The Qindao University, Qindao, 266001 China; 4 LanZhou University of Technology, School of Life Science and Engineering, Lanzhou, 730050 China

**Keywords:** Oligomannuronate, Type 2 diabetes, Islet amyloid polypeptide, JNK activation, Mitochondrial dysfunction, RINm5F cells

## Abstract

**Background:**

Oligomannuronates (OM) are natural products from alginate that is frequently used as food supplement. The aim of this study was to investigate the in vitro protective effects of OM on RINm5F cells against human Islet amyloid polypeptide (IAPP) induced mitochondrial dysfunction, as well as the underlying mechanisms.

**Methods:**

In the present study, we obtained several kinds of OM with different molecular masses, and then we used RINm5F cells as a model to elucidate the involvement of JNK signal pathway in hIAPP-induced mitochondrial dysfunction in pancreatic beta cells, and the protective effects of OM are associated with its ability to attenuate the mitochondrial dysfunction.

**Results:**

Our results demonstrated that human IAPP induced mitochondrial dysfunction, as evidence by loss of ΔΨm and ATP content, and decrease in oxygen consumption and complex activities, was accompanied by JNK activation, changes in the expressions of Bcl-2 and Bax proteins, release of cytochrome c (Cyto-c) and apoptosis inducing factor (AIF) from mitochondria into cytosol. Interestingly, the human IAPP induced damage in RINm5F cells were effectively restored by co-treatment of OM. Moreover, JNK activation was required for the OM mediated changes in RINm5F cells.

**Conclusions:**

OM prevented mitochondrial dysfunction induced by human IAPP in RINm5F islet cells through JNK dependent signaling pathways.

## Background

Islet formation is a hallmark of type 2 diabetes occurring in most patients [1, 2], which is associated with decrease beta cell function and mass. Islet amyloid polypeptide (IAPP) is a major component of the amyloid deposits. Human IAPP (hIAPP) is a peptide hormone composed of natural unstructured 37 residues that are commonly stored and secreted from the beta cell secretory granules of islets [1, 3]. hIAPP contains critical structure for β-pleated sheet formation, which has propensity to form amyloid fibrils [4]. Gradual loss of insulin-producing islet beta cell mass in type 2 diabetic patients leads to increased islet amyloid deposits. Moreover, synthetic hIAPP fibrils and oligomers induced apoptosis in human or rat islet beta cells. In the hIAPP transgenic mouse model, hyperglycemia causes an increase in amyloid formation and a decrease in beta cell mass [5, 6]. Some researches indicate that the cytotoxicity of hIAPP according with endoplasmic reticulum stress, oxidative stress and mitochondrial dysfunction [5, 7].

It is well known that mitochondria have important functions in storing energy, regulating metabolism and cell growth pathways. Previous reports suggested that metabolic dysregulation and insulin secretory failure might be due to mitochondrial dysfunction in the pathogenesis of type 2 diabetes [8, 9]. Moreover, mitochondria participate in the control of islet beta cell mass. Several reports demonstrated that apoptosis due to mitochondrial dysfunction was especially apparent in pancreatic beta cells [7, 10, 11]. Besides, another central pathway to regulate beta cell growth and apoptosis is the c-Jun N-terminal protein kinase (JNK) pathway, which is activated in response to several stress stimuli, such as high glucose [12], ER stress [13, 14], and hIAPP [15–17]. Recent investigations on the interplay between JNK pathway and mitochondria is focused on, and results revealed that the functional interactions of JNK and mitochondria is complicated and remains to be built up [18–22], and moreover, the link between mitochondria function and JNK pathway in hIAPP-induced beta cell has not been reported.

Alginate, is a kind of acidic linear polysaccharide which consists of β-d-mannuronic acid and α-l-guluronic acid [23]. Alginate oligosaccharide and their derivatives have been widely used in the food, cosmetics and pharmaceutical industries [23, 24]. Recently, low molecular weight alginate has attracted lots of scientific interest due to their bioactivity of plant growth promotion [25], anti-tumor [26, 27], and neuron protection [28, 29]. Moreover, some alginate-derived OM showed an outstanding activity in glucose and lipid metabolism in C2C12 skeletal muscle cells [30]. But there is no detailed information about the effects of OM on the pancreatic beta cells, no mention the comparative research in the relationship between the effects and OM with different molecular masses.

In the present study, we obtained several kinds of OM with different molecular masses, and then we used RINm5F cells as a model to elucidate the involvement of JNK signal pathway in hIAPP-induced mitochondrial dysfunction in pancreatic beta cells, and the protective effects of OM are associated with its ability to attenuate the mitochondrial dysfunction.

## Materials and methods

### Materials and reagents

Polymannuronic acid (M-block) with weight-average molecular weight (*M*w) of 21.0 kDa was provided by Lantai Pharmaceutical Company (Qingdao, China). RPM1640 medium, fetal bovine serum (FBS), penicillin, and streptomycin were purchased from Gibco (Grand Island, NY). Anti-phospho JNK (#4668), anti-Cyto c (#4280), anti-Bax (#5023), anti-Bcl 2 (#3498) and anti-AIF (#5318) were from Cell Signaling Technology; other chemicals that were not specifically mentioned here were purchased from Sigma.

### Preparation of OMs and analytical methods

M-block was dissolved with dilute ammonia water in a Pyrex tube (pH = 5) and hydrolyzed under microwave irradiation (1600 W, CEM corporation, Matthews, North Carolina, USA) at different conditions as previously described [31]. Four OMs were prepared at 2% (w/v), 130 °C, 20 min; 5% (w/v), 120 °C, 5 min; 5% (w/v), 100 °C, 20 min and 8% (w/v), 100 °C, 10 min,respectively. The hydrolysates were decolorized by activated carbon and filtered by 0.22 μm cellulose membrane, then freeze-dried to obtain OMs with different *M*w compounds.

The relative weight-averaged molecular weight (*M*w) of OMs made by microwave degradation were determined by high performance gel permeation chromatography combined with multi-angle laser light scattering (HPGPC-MALLS) on an Agilent 1260 chromatographic instrument. The OMs was dissolved in 0.1 mol/L Na_2_SO_4_ elution solvent at a concentration of 5 mg/mL. Then 100 μL of the sample was applied to a TSK gel G3000PWXL column (7.8 mm × 300 mm, Tosoh, Japan) with a flow rate of 0.6 mL/min. The temperature of column was maintained at 35 °C and the signal was detected by G1362A refractive index detector (RID) and MALLS (Dawn Heleos-II, Wyatt technologies, USA). The *M*w of each sample was calculated by Astra 5.3.4.20 software.

The total sugar content of OMs was determined according to the method of Dubois et al. using d-glucuronic acid as the standard [32]. The amount of uronic acids was determined using the carbazole reaction method described by Bitter and Muir, and d-glucuronic acid was used as the standard [33].

### Infrared spectral and NMR analysis

For FTIR analysis, the dried samples (1–2 mg) were mixed with 100 mg dried KBr and pressed to a transparent pellet and then analyzed with a Nicolet Nexus 470 FTIR spectrometer (Thermo Electron) under dry air at 400–4000 cm^−1^. And For ^1^H-NMR and ^13^C-NMR analyses, the sample was dissolved in D_2_O (99.96%) and freeze-dried twice to replace all exchangeable protons with deuterium. Spectra were acquired at 25 °C using JNM-ECP 600 MHz equipment (JNM-ECP 600, Jeol, Japan). Chemical shift values were calibrated using acetone-*d*6 as an internal standard. The data were processed using the MestReNova software.

### Cell culture and treatments

RINm5F cells, from American Type Culture collection (ATCC), were cultured in RPMI 1640 medium supplemented with 10% (v/v) fetal bovine serum, 2 mM l-glutamine, 100 U/mL penicillin, and 100 μg/mL streptomycin, at 37 °C in a humidified (5% CO_2_, 95% air) atmosphere. All experiments were performed using cells between passages 21 and 30.

For peptide treatment, lyophilized hIAPP (Calbiochem) was dissolved in 80% hexafluoroisopropanol (HFIP, Sigma-Aldrich) to a concentration of 521 μM, filtered over 0.2 μm filters (Millipore), and stored at − 20 °C until use. hIAPP aggregates were prepared by diluting hIAPP to 6.5 μM in 10 mM phosphate buffer, pH7.4, 1.0% HFIP, and by incubating the mixture at 25 °C for 30 min before use.

Cells were pre-incubated with various concentrations of OMs for 48 h, and then remove the OMs and change for the fresh medium. Meanwhile, the hIAPP aggregates solution was applied to cultured cells at a final concentration of 250 nM for 24 h.

### MTT assay

RINm5F cells seeded in 96-well microplate were cultured at 37 °C in a humidified atmosphere for 72 h, and then the cells were exposed to different treatments for different periods of time. After incubation, 20 μL/well of MTT solution (5 mg/mL in PBS buffer) was added and incubated for 4 h. The medium was aspirated and replaced with DMSO to dissolve the formazan salt. The color intensity of the formazan solution, which reflects the cell growth condition, was measured at 570 nm using a microplate spectrophotometer.

### Measurement of mitochondrial membrane potential (MMP, ΔΨm)

Determination of ΔΨm was carried out using the ratiometric dye JC-1 (5,5′,6,6′- tetrachloro-1,1′,3,3′-tetraethyl-benzimidazolyl-carbocyanine iodide), which is a dual emission potential-sensitive probe [34]. Freshly prepared cells were incubated with 10 μg/mL of JC-1 for 30 min at 37 °C, washed twice with PBS, and then analyzed by a dual-wavelength/double-beam recording spectrophotometer (Ex490nm, Em530 and Ex525nm, Em590). ΔΨm = A525-A590/A490-A530 (Flex Station384, Molecular Devices, USA).

### Detection of intracellular Reactive Oxygen Species (ROS)

The production of cellular ROS was measured by 2′,7′-dichlorofluorescin diacetate (DCFH2-DA) oxidation [35]. Freshly prepared cells were incubated with 2 μM DCFH2-DA for 30 min at 37 C, and then washed with PBS buffer, and the fluorescence was measured at 485 nm excitation and 535 nm emission recording spectrophotometer (Flex Station 384, Molecular Devices, USA).

### Determination of ATP content

The intracellular ATP content was determined using a bioluminescence somatic cell assay kit according to the manufacturer’s instructions. After various treatments, cells were lysed by 0.5% Triton X-100 in 100 mM glycine buffer, pH 7.4. Intracellular ATP levels were assayed with an ATP bio-luminescence assay kit (Sigma) based on the luciferase-catalyzed oxidation of d-luciferin [36]. The number of viable cells was counted with trypan blue using a hemacytometer. ATP level was expressed as femtomoles per cell.

### Mitochondrial respiration

Oxygen consumption by intact cells was measured as described [37]. After treatment, the islet beta cells were washed in KRH buffer plus 1% BSA. Cells from each condition were divided into triplicate aliquots and measured in a BD Oxygen Biosensor System plate (BD Biosciences). Plates were sealed and ‘read’ on a fluorescence spectrometer (Molecular Probes, Eugene, OR, USA) at 1 min intervals for 60 min at an excitation wavelength of 485 nm and emission wavelength of 630 nm. We have used 5 × 10^5^ cells in the assay. The oxygen consumption rate of cells generally follows Michaelis–Menten kinetics with respect to oxygen concentration. V_max_ is the maximum consumption rate.

### Activity of mitochondrial complexes I, II and III

Adipocytes were cultured in 100 mm plates and washed in PBS. Following addition of trypsin, the cells were pelleted by centrifugation at 300 g for 5 min at 4 °C. All of the subsequent steps were performed on ice. The resulting pellet was then resuspended in mitochondrial isolation buffer (215 mM mannitol, 75 mM sucrose, 0.1% BSA, 1 mM EGTA, 20 mM HEPES, pH 7.2) and homogenized on ice with a glass homogenizer. The mitochondria were then purified by differential centrifugation at 1300 g for 5 min to pellet unbroken cells and the nuclei. The supernatant fraction was then centrifuged at 13 000 g for 10 min to pellet the mitochondria. The pellet was resuspended in EGTA-free isolation buffer. Briefly, complex I activity was assayed by monitoring the decrease of NADH at 340 nm. Final concentration of mitochondrial protein was 30 mg/mL. Reaction was started by adding 200 mM NADH and scanned at 340 nm for 3 min. Rotenone (3 mM) was added into the reaction system as blank control. Complex II was assayed with mitochondria (final concentration 30 mg/mL), and the reaction was started with 10 mM succinate and scanned at 600 nm for 2 min. Complex III activity was measured in a mixture containing 250 mM sucrose, 1 mM EDTA, 50 mM K phosphate, pH adjusted to 6.5 to reduce autooxidation of reduced coenzyme Q1 (CoQ1), 2 mM KCN, 50 mM Cyto-c, 0.1% BSA, and the reaction was initiated by 20 mg/mL mitochondria and 50 mM reduced CoQ1, recording the increase of absorption at 550 nm for 2 min [38].

### Western blot analysis

To obtain cytosolic and mitochondrial fractions, the cells were incubated with buffer (20 mM Hepes, 1 mM EDTA, 10 mM KCl, 1.5 mM MgCl_2_, 1 mM EGTA, 1 mM dithiothreitol, 250 mM sucrose, aprotinin, leupeptin and pepstain 2 mg/mL each, pH7.5) on ice for 30 min. After the cells were disrupted in a glass dounce homogenizer by optimized gentle strokes, homogenates were centrifuged at 1000 g for 10 min at 4 °C to remove nuclei. The supernatant were further centrifuged at 12,000*g* at 4 °C for 30 min to separate the miochondria and cytosol fractions. To prepare whole cell lysates, the cells were harvested and incubated with lysis buffer by votex. After centrifugation at 13,000*g* at 4 °C for 30 min to separate the cellular debris, the supernatant was collected and stored at − 80 °C until use. The protein concentrations were determined using BCA protein assay kit according to the manufacture’s instructions.

Cell lysates (10 mg protein per lane) were subjected to 10% SDS-PAGE, then transferred to nitrocellulose membranes and blocked with 5% non-fat milk/Tris-buffered saline with Tween (TBST) for 1 h at room temperature. Membranes were incubated with primary antibodies directed against β-actin (1:10 000), p-JNK, Bax, cyto-C Bax, Bcl 2 and AIF in 5% milk/TBST at 4 °C overnight. After washing with TBST three times, membranes were incubated with horseradish peroxidase-conjugated secondary antibody for 1 h at room temperature. Western blots were developed using ECL (Roche Mannheim, Germany) and quantified by scanning densitometry [39]. Relative protein expressions determined by Western blotting were normalized to β-actin expression. Results were presented as percent control.

### Statistics

All values are expressed as mean ± S.E.M. Statistical significance was determined by using one-way ANOVA with Bonferroni’s post hoc tests between the two groups. The criterion for significance was set at P < 0.05.

## Results

### Characterization of OMs

**Fig. 1:**
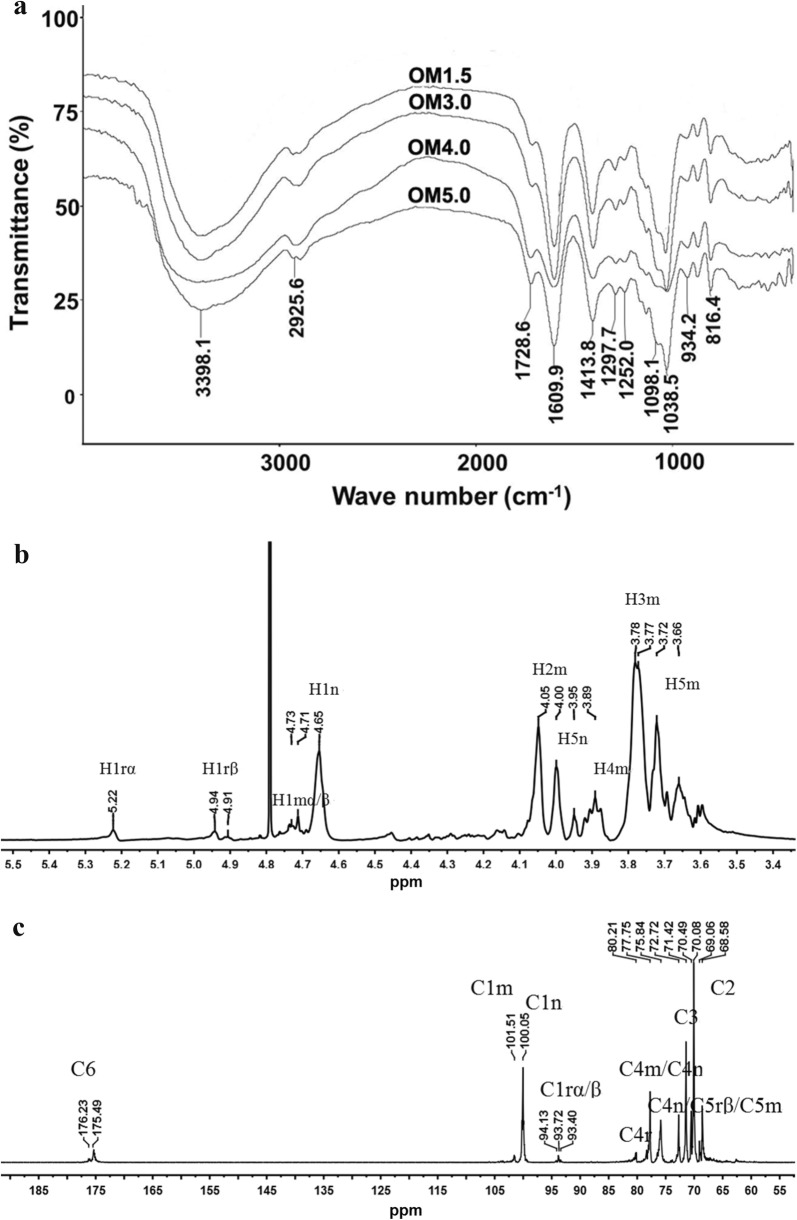
The FTIR and 1D-NMR analysis. **a** FTIR spectrum of OMs. **b**^1^H-NMR spectrum of OM3.0. **c**^13^C -NMR spectrum of OM3.0

The *M*w of OMs was 1678 Da for OM1.5, 3317 Da for OM3.0, 3910 Da for OM4.0, 5266 Da for OM5.0, respectively (Table 1). And the FT-IR spectrums of OMs are shown in Fig. 1a. The data revealed the bands at around 3398 cm^−1^ were assigned to symmetric stretching vibration of the hydroxyl group. The band at around 1729 cm^−1^ was attributed to C=O stretching of carboxyl. The asymmetric and symmetric stretching of carboxylate vibrations appeared at 1610 cm^−1^ and 1414 cm^−1^. The band at around 1098 cm^−1^ (C–O–C stretching) and 1039 cm^−1^ (C–O stretching) were the characteristic absorption bands of polysaccharide structure. The band at around 934 cm^−1^ was the characteristic asymmetric stretching vibration of pyranose rings. The bands appeared at 816 cm^−1^ were mannopyranuronic acid. Therefore the products obtained from depolymeration of the PM remained mannuronate structure.

**Table 1:** The relative weight-average molecular weight (*M*w), total sugar content and uronic acid content of OMs

Samples	*M*_w_ (Da)	D	Total sugar (%)	Uronic acid (%)
OM1.5	1678	1.02	93.27 ± 1.39	88.58 ± 1.88
OM3.0	3317	1.21	93.47 ± 0.80	88.83 ± 0.92
OM4.0	3910	1.34	94.21 ± 1.64	91.46 ± 0.93
OM5.0	5266	1.40	95.17 ± 2.46	93.28 ± 1.08

The structures of MOs were further characterized by NMR spectroscopy. To take OM3.0 as an example, the chemical shifts of anomeric protons were assigned according to ^1^H-NMR spectrum. As shown in Fig. 1b, the apparent single peak at 5.22 ppm and double peak at 4.91–4.94 ppm corresponded to the reducing-end H1 and H1β signals respectively. The signal at 4.77 ppm was observed for the middle ring C1 H signal. The peak at 4.65 ppm was seen for the C1 proton of non-reducing-end. The signals between 3.6 ppm and 4.0 ppm were assigned to H2, H3, H4 and H5 of the reducing end, middle residue and non-reducing end. The 600 MHz ^13^C NMR spectra of OM3.0 are given in Fig. 1c. Chemical shifts were unambiguously assigned. A single peak at 175.49–176.23 ppm was seen for the C6 of pyranose rings. The peak at around 94 ppm corresponded to the C1 signals of the reducing end. Peaks at 101.51 ppm and 100.05 ppm were assigned to C1 of non-reducing end and the middle ring, respectively. The signal appeared at 77.75 ppm was assigned to C4r and C4m. The signal appeared at 75.84 ppm was seen for the C4n/C5rβ/C5m. The peak at 72.72 ppm was assigned to the resonance of C5n. The signals appeared around 69–72 ppm were attributed to C3 and C2 of pyranose rings, respectively.

### Cell toxicities of OMs

**Fig. 2:**
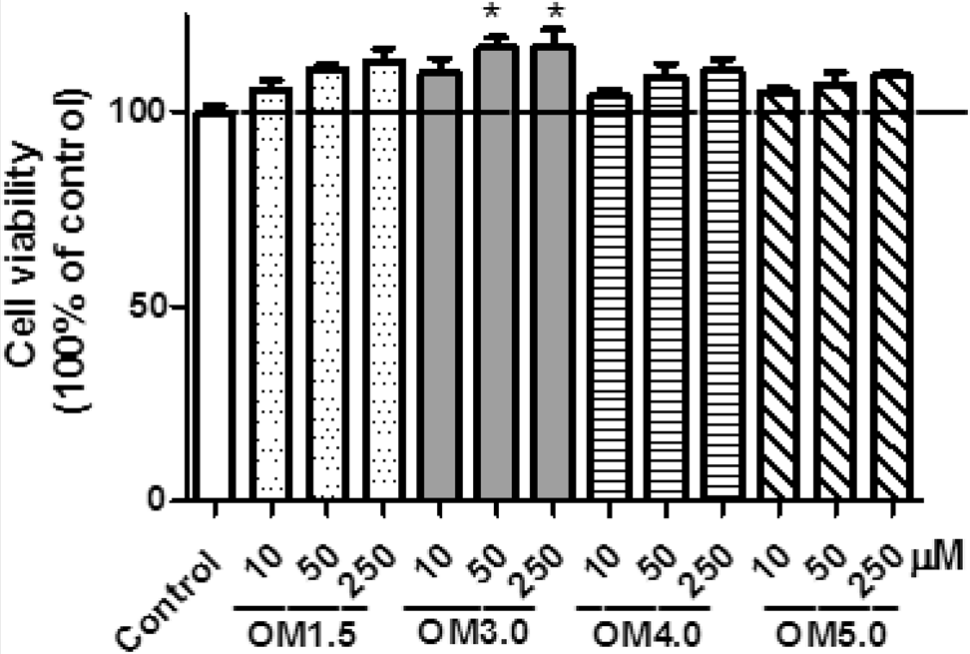
The cytotoxicities of OMs on RINm5F cells. Cells were incubated with indicated OMs at indicated concentrations for 72 h. The cell viability was evaluated by MTT assay as described in materials and methods. Values are mean ± S.E.M of four replicates. *P < 0.05 versus the IAPP model

As shown in Fig. 2, treatment of RINm5F cells with different concentrations of OMs indicated as OM1.5, OM3.0, OM4.0, OM5.0, tended to increase the cell viability determined by MTT method, which demonstrated that OMs had no toxic effect on RINm5F cells within the ranged concentrations of 0–250 μM, and these results were in line with our previous report in C2C12 skeletal muscle cells [30]. Meanwhile, the OM3.0 treatment could significantly increase the cell viability of RINm5F cells at levels of 50–250 μM. In addition, there was no obvious increase in the MTT assay of RINm5F cells induced with OMs at the concentration of 250 μM as compared with 50 μM. So we choose the 10 and 50 μM to continue our ΔΨm detection.

### Effect of OMs on mitochondrial membrane potential (MMP)

**Fig. 3:**
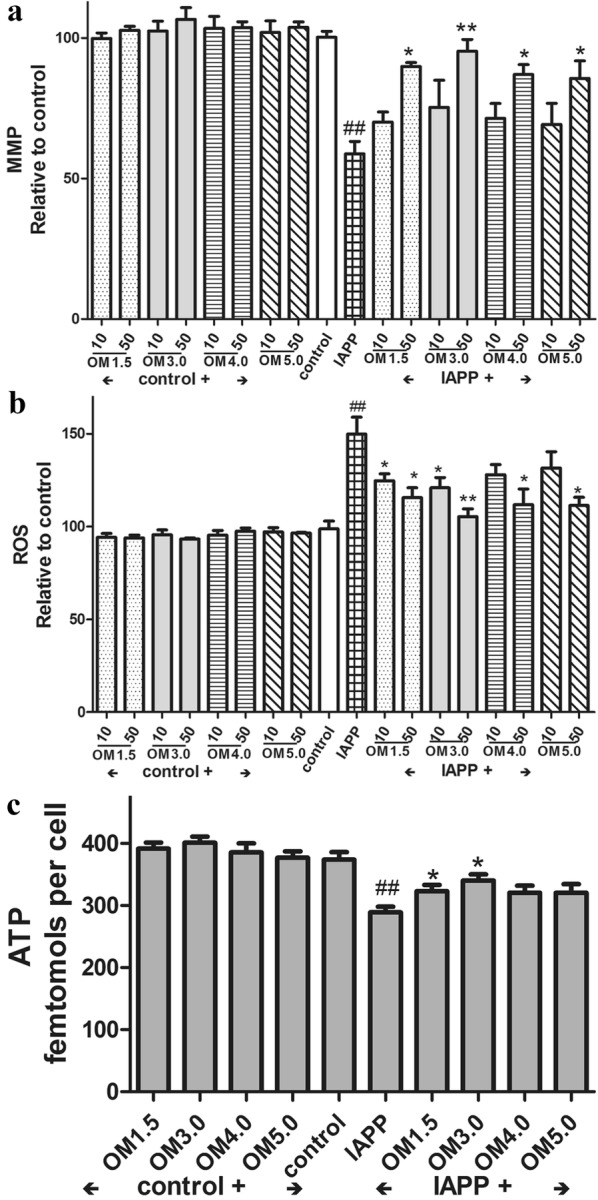
Influence of OMs on the ΔΨm, ROS production and ATP content. Cells were incubated with OMs at indicated concentrations for 48 h, and then induced with IAPP (250 nM) for 24 h. After treatment cells were washed and incubated with JC-1 for ΔΨm determination (**a**); or washed and incubated with 2 μM DCFH2-DA for ROS production assay (**b**); or washed and the ATP contents were determined by a luminescence assay kit based on the luciferase-catalyzed oxidation of d-luciferin (**c**). Values are mean ± S.E.M of the results from at least three independent experiments. ^##^P < 0.01 versus control; *P < 0.05, **P < 0.01 versus the IAPP model

As shown in Fig. 3a, pre-treatment of RINm5F cells with OMs at different concentrations as indicated could obviously inhibit the reductions of ΔΨm in RINm5F cells induced by IAPP as determined by JC-1 assay. And among the OMs with different molecular weights from 1.5 kDa to 5.0 kDa, the OM3.0 with molecular weight of 3.0 kDa tends to be the best one. Meanwhile, in the absence of IAPP additon, OMs had no effect on RINm5F cells within the ranged concentrations of 10–50 μM.

Then the ROS production was examined and the results were shown in Fig. 3b, IAPP treatment at 250 nM for 24 h led to significantly increase in ROS production in RINm5F cells, and pre-treatment of the cells with the OMs could distinctly inhibit ROS production induced by IAPP.

Meanwhile, OMs also had no effect on RINm5F cells within the ranged concentrations of 10–50 μM. In addition, it is obviously that the optimal concentration of OMs to exert their protective effects on RINm5F cells induced by IAPP is 50 μM, which was in line with the results of JC-1 assay. And similarly, the OM3.0 was the best one to protect the RINm5F cells from damage induced by IAPP.

Based on the data shown in Fig. 3a, b, the effective concentration of OMs to protect RINm5F cells was optimized at 50 μM. Thus, the RINm5F cells were then treated with OMs at concentrations of 50 μM. Figure 3c showed that the ATP levels was significantly reduced by IAPP addition, and the OMs pre-treatment at 50 μM could obviously prevent the islet beta cells from decrease in ATP content in RINm5F cells stimulated by IAPP. The OM3.0 as well tended to be the most effective one to protect RINm5F cells.

### Effect of OMs on mitochondrial functions

**Fig. 4:**
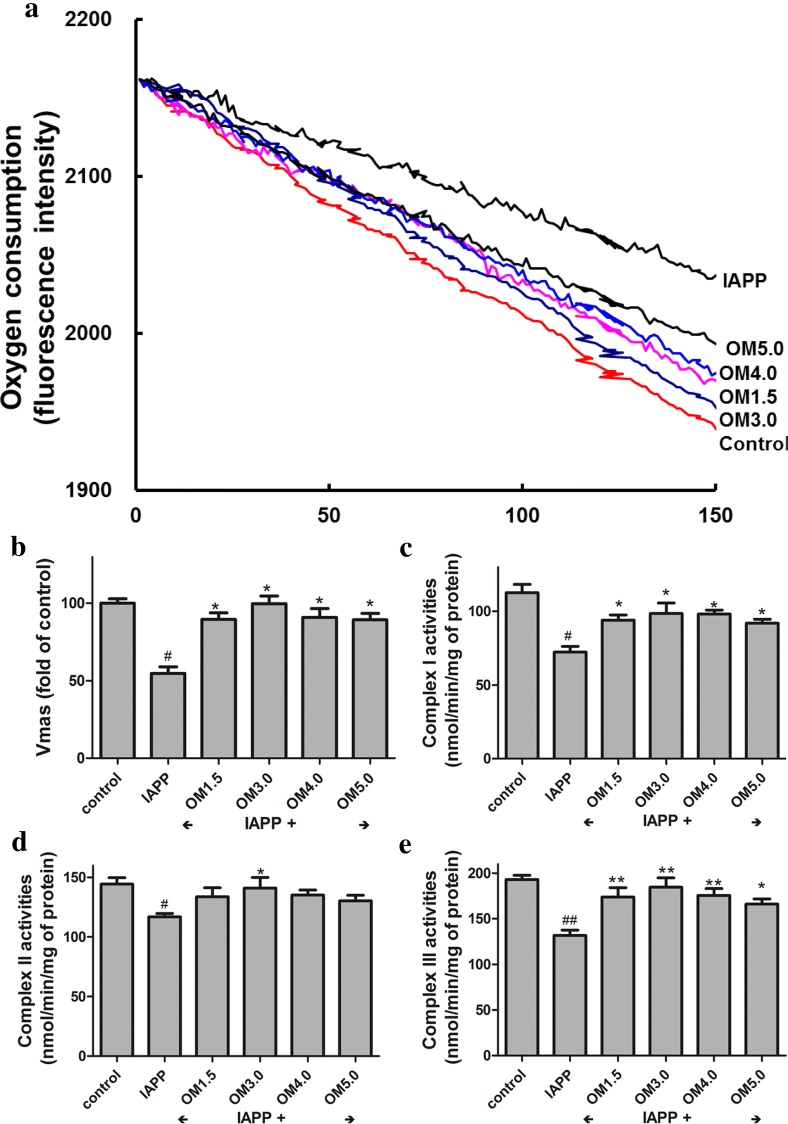
OMs inhibited IAPP induced mitochondrial dysfunction in RINm5F cells. The Islet β cells were seeded in 6-well plates and treated with OMs at 50 μM for 48 h, and then induced with IAPP (250 nM) for 24 h. After treatment, cells were presented for oxygen consumption in that equal volumes of cells were separated into aliquots in wells of a 96-well BD Oxygen Biosensor plate. Fluorescence in each well was recorded over time. **a** Representative oxygen consumption curves. **b** Quantitative changes in the respiratory rate of the islet β cells calculated by determining the kinetic measurements. Or the mitochondrial of RINm5F cells were isolated for detection of complex activities. **c** Mitochondrial complex I activity assay. **d** Mitochondrial complex II activity assay. **e** Mitochondrial complex III activity assay. Values are mean ± S.E.M of the results from at least three independent experiments. ^##^P < 0.01 versus control, *P < 0.05 versus the IAPP model

We next measured if the OMs induced increase in ΔΨm and ATP content were correlated with enhanced mitochondrial functions. Figure 4 indicated that the OMs pre-treatment at 50 μM could significantly inhibit the decrease of oxygen consumption (Fig. 4a, b) in the RINm5F cells induced by IAPP treatment, which was associated with the up-regulation of mitochondrial complex I (Fig. 4c), complex II (Fig. 4d) and complex III (Fig. 4e) activities by OMs pre-incubation in the beta cells followed by IAPP induction. All these results presented in Figs. 3, 4 showed that the OMs, especially the OM3.0 could distinctly enhance mitochondrial functions, which were associated not only with increased ΔΨm, ATP levels, oxygen consumption and complex I–III activities, but also with decreased ROS production.

### Effect of OM3.0 on key-protein expressions of JNK signaling pathway

**Fig. 5:**
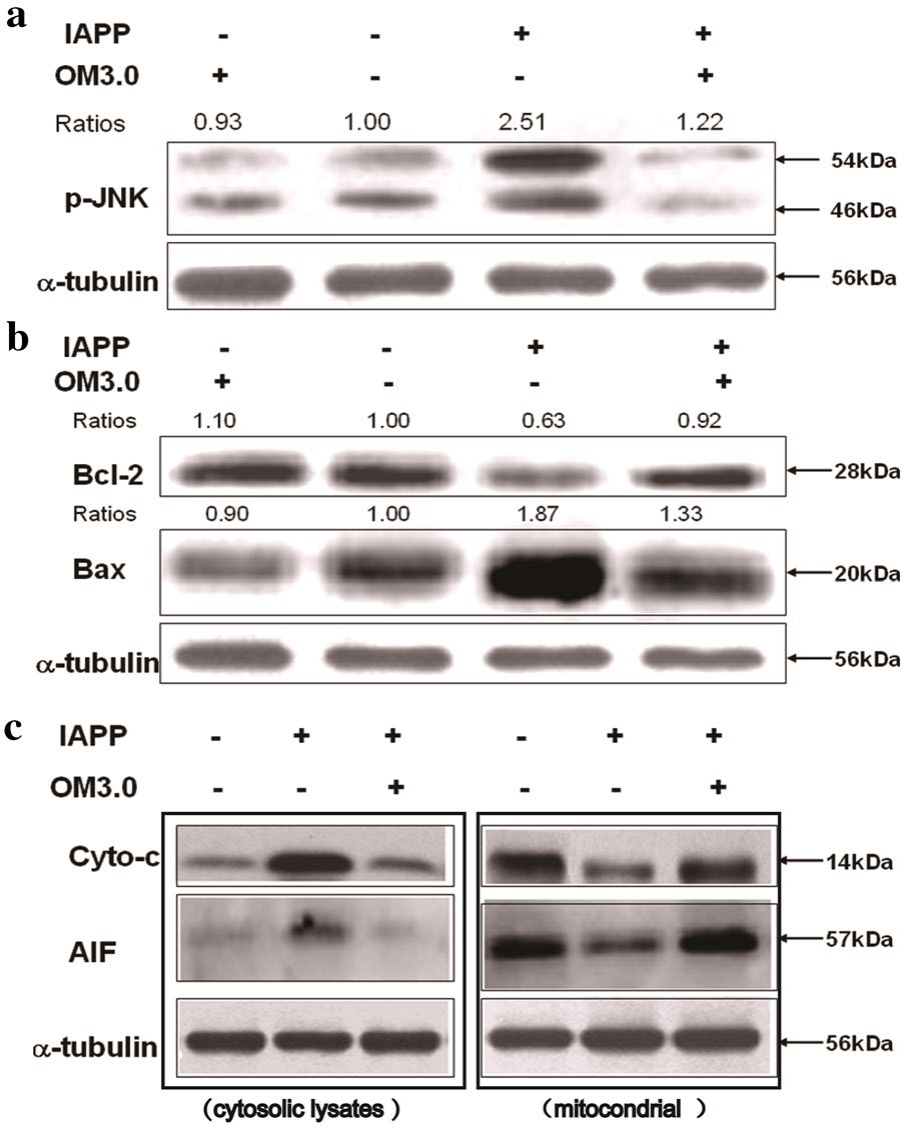
OM3.0 suppressed JNK activation and cell apoptosis in IAPP induced islet β cells. RINm5F cells in 6-well plates were treated with OMs at 50 μM for 48 h, and then induced with IAPP (250 nM) for 24 h. **a** Inhibition of JNK activation by OM3.0 treatment. Cell lysates were subjected to western blot analysis of phosporylated JNK. α-tubulin was used as an internal control. **b** Effect of OM3.0 on protein expression of Bcl-2 and Bax. Cell lysates were subjected to westrern blot analysis of Bcl-2 and Bax, α-tubulin was used as an internal control. **c** OM3.0 prevented IAPP induced changes of Cyto-c and AIF release from mitochondria into cytosol. The treated cells were separated into cytosolic and mitochondrial fractions. Cell lysates were subjected to western blot analysis. Changes in levels of protein expression (shown as ratios) were calculated based on levels in corresponding untreated cells, which were set at unity. Data represent similar results from three independent experiments

Figures 2, 3 and 4 demonstrated that the optimal effect of stimulation was found to be at 50 μM, and OM3.0 was the most effective compound among the four kinds of oligosaccharides with different molecular weight. Thus, the RINm5F cells were then treated with OM3.0 at concentrations of 50 μM to continue our experiments. It was reported that JNK signaling pathway play important roles in the damage and dysfunction of islet beta cells [40]. We performed the western blotting assays for determination of JNK activation and expression of cell apoptotic regulating proteins and factors. As shown in Fig. 5, the p-JNK level was significantly up-regulated by IAPP induction for 24 h at 250 nM (Fig. 5a), the protein expressions in Bcl-2 and Bax were also obviously changed by IAPP induction (Fig. 5b), which were correlated with the increased cytosolic Cyto-c content and AIF levels, and the elevated cytosolic Cyto-c and AIF release were accompanied by the decreased mitochondrial Cyto-c and AIF levels (Fig. 5c). After IAPP treatment of cells, it promotes the release of cytochrome C from mitochondria to the cytoplasm, which is also a sign of apoptosis. It is obvious that the OM3.0 pre-treatment could distinctly prevent the RINm5F cells from the JNK activation and apoptosis induced by IAPP addition.

### JNK activation was required for OM3.0 mediated effects

**Fig. 6:**
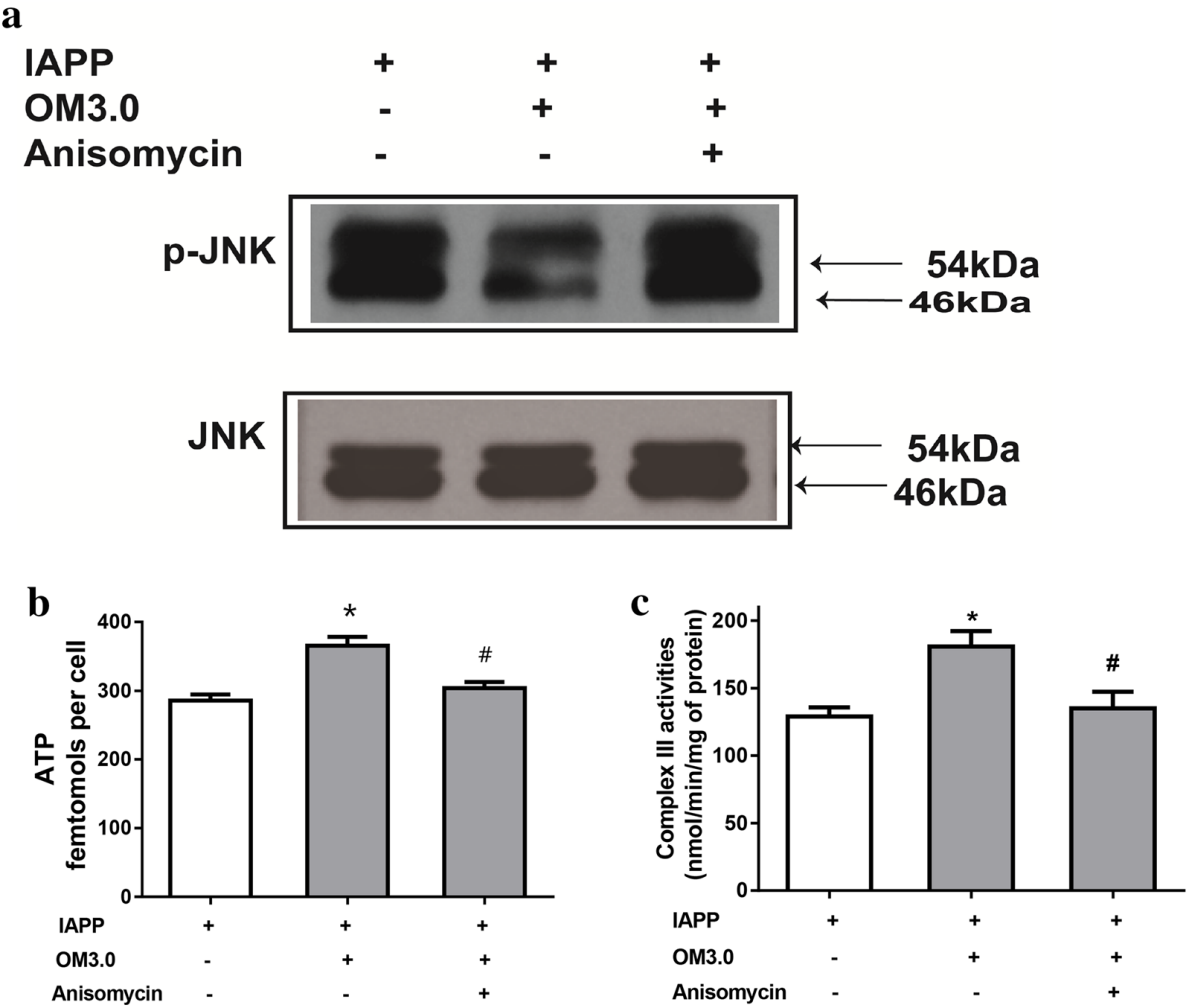
Requirement of JNK activation in OM3.0 mediated changes in IAPP induced RINm5F cells. Cells were pretreated with OM3.0 and IAPP for 16 h and then with or without anisomycin as described. Protein isolates were subjected to **a** western blot analysis of JNK activation, **b** determination of complex III activity, and **c** assay of ATP content. Change in level of JNK phosphorylation (shown as ratio) was calculated based on level in corresponding untreated cells, which were set at unity. Values are mean ± S.E.M of the results from at least three independent experiments. ^##^P < 0.01 versus control, *P < 0.05 versus the IAPP model, ^∇^P < 0.05 versus the anisomycin treatment

To test if the increased mitochondrial function and reduced apoptosis were the results of the inhibited JNK pathway by the OM3.0 treatment, we decided to use the anisomycin to stimulate JNK activation to understand the relationship between them. As shown in Fig. 6a, OM3.0 dramatically inhibited the activation of JNK as determined by western blotting for p-JNK expression, which were in agreement with Fig. 5a. But the inhibitory effect of OM3.0 on JNK activation was abrogated by anisomycin treatment, a specific JNK activator. Meanwhile, the increase of mitochondrial complex III activities and cellular ATP content stimulated by OM3.0 were both significantly reversed by anisomycin treatment (Fig. 6b, c). All these results demonstrated that the OM could prevent mitochondrial dysfunction induced by IAPP in RINm5F islet cells through JNK dependent signaling pathway.

## Disscussion

A critical feature of diabetic pathogenesis is the presence of amyloid, that are associated with the death of insulin secreting beta cells. These amyloid deposits are composed primarily of protein fibers formed from IAPP (also known as amylin). IAPP is a 37 residue, natively unstructured peptide hormone that is co-packaged with insulin in the secretory granules of the beta cells [4, 6]. In the present study, we demonstrated for the first time that mitochondrial dysfunction induced by hIAPP was dependent on the JNK signaling pathway in pancreatic beta cells. Our results showed that disruption of mitochondrial function in hIAPP induced RINm5F cell, as evidence by loss of MMP, suppressed mitochondrial complex activities, ATP depletion, decreased respiratory consumption, elevated apoptotic protein expression and Cyto-c release, was mediated by the JNK activation. Interestingly, these hIAPP-induced changes were significantly attenuated by OMs treatments, which were partially due to its ability to prevent mitochondrial dysfunction through a JNK mediated signaling pathway.

Recent years, lot of poly-/oligosaccharides have been investigated and showed anti-diabetic activities, for example, hypoglycemic activities have been revealed for chito-oligosaccharides, and oligosaccharides from two kinds of fungi [41–44]. But no information about the beneficial effect of OMs on the hIAPP-induced pancreatic beta cells. Due to the commercially wide use of OMs, we obtained several kinds of OMs with different molecular mass by microwave degradation, and compared their protective effects on the MMP loss induced by hIAPP in RINm5F cells. In the present work, several water-soluble OMs were prepared by microwave-irradiation method. The molecular weights of OMs were about 1.5–5.0 kDa, as determined by HPGPC-MALLS. And the results showed that OMs, especially for the molecular weight of 3.0 kDa, could effectively inhibit the MMP loss in hIAPP-treated RINm5F cells. In our previous study, we also found that the OM-derived chromium (III) complexes with the molecular weight of around 3.0 kDa significantly stimulated insulin sensitivity in C2C12 skeletal muscles [30]. These data demonstrated an optimized concentration of OM around 3.0 kDa to exert its bioactivity on skeletal or pancreatic islet beta cells, which could share some insight for its potential in industrial applications.

The mitochondrion of pancreatic beta cell is a key organelle for control of insulin secretion. Several published studies revealed that beta cell apoptosis due to mitochondrial dysfunction was especially apparent in pancreatic beta cells [8, 9]. The mitochondrial respiratory chain is a major source of ROS whose overproduction of ROS will further impair mitochondrial electron transport to cause more ROS release. ROS has been highlighted as initiators for hIAPP-induced cytotoxicity in pancreatic beta cells. Previous findings suggested that over-production of ROS was involved in hIAPP-induced beta cell toxicity [1, 13, 45]. Moreover, ROS are known to affect MMP, and the loss of MMP induces release of mitochondrial apoptogenic factors, such as Cyto-c and AIF [10, 11]. Once in the cytol, Cyto-c binds to adaptor molecule apoptotic protease activating factor I (Apaf-1) in the presence of ATP and activates caspase-9, which in turn activates caspase-3 and cleaves downstream PARP, and eventually leads to cell apoptosis [46]. In contrast to Cyto-c, AIF mediated the apoptotic cell death trough a caspase-independent pathway [47]. In the present study, we found that hIAPP treatment induced intracellular ROS generation in RINm5F cells accompanied with damages in mitochondrial function, including loss of MMP, depletion of ATP, release of apoptogenic factors such as Cyto-c and AIF, increased expression of Bax, and decreased expression Bcl-2, which was attenuated by co-treatment with OMs. Meanwhile, the JNK activation and downstream targets were also down-regulated by OMs treatments in pancreatic beta cells. These results suggest that OM is capable of scavenging free radicals and protects RINm5F cells against hIAPP-induced mitochondrial damage, which was associated with the activation of JNK signaling pathway.

JNK signaling has an established role in the regulation of cell apoptosis through the activation of transcription factors and phosphorylation of apoptosis-related proteins [40, 48, 49]. The JNK signaling pathway has been reported to affect members of the Bcl-2 family. For example, JNK not only can inactivate anti-apoptotic Bcl-2 proteins but also can activate the mitochondrial translocation of Bax [50]. But conversely, there is growing evidence that JNK signaling can induce pro-survival pathways in certain cell types, including neurons, T cells and B lymphocytes [51, 52]. Thus, the role of JNK is complex in the mediation of distinct cellular response depending on cell contents. In addition, different concentrations of hIAPP could result in different cellular responses, for example, the activation of NADPH-oxidase induced by hIAPP at 60 nM was converse to the concentration at 500 nM [53]. And recent researches were focused on the molecular or signal changes induced by hIAPP at μM levels [1, 6, 7, 16, 40, 54]. And in RINm5F cells, activation of JNK was associated with hIAPP-induced cell damage [15, 54]. In the present study, we firstly found that hIAPP at 250 nM could effectively stimulate the activation of JNK in RINm5F cells. In support of the pro-apoptosis functions of JNK in the presence of hIAPP, our results also demonstrated that the protective effects of OM on beta cells were partially mediated by suppressing the activation of JNK pathway.

The signal interactions of JNK with mitochondria were complicated as reported [18–22], in the present study, we demonstrated for the first time that the mitochondrial dysfunction induced by hIAPP was dependent on the activation of JNK. Meanwhile, the stimulation of JNK by specific activators also obviously abrogated the beneficial effect of OM on the hIAPP-induced mitochondrial damage in RINm5F cells, indicating the prevention of mitochondrial damage in IAPP-treated RINm5F islet cells by OM treatment was through a JNK dependent signaling pathway.

## Conclusions

In this study, our results provide strong evidence that hIAPP-induced mitochondrial dysfunction in RINm5F islet beta cells through was through activation of JNK signaling pathway. Moreover, treatment with OM effectively attenuated hIAPP-induced loss of MMP, ATP depletion, reduced mitochondrial complex activities, decreased Bcl-2, increased Bax levels protein and release of Cyto-c, these effects were in part mediated by the activation of JNK. The present work enhances our understanding of the molecular mechanisms of hIAPP-induced pancreatic mitochondrial dysfunction and indicates the potential therapeutic role of OM in type 2 diabetes by preventing hIAPP-induced loss of pancreatic beta cells.

## Data Availability

The datasets used during the present study are available from the corresponding author upon reasonable request.

## Abbreviations

MMP: Mitochondrial membrane potential
JC-1: J-Aggregate forming lipophilic cation 55′,6,6′-tetrachloro-1,1′,3,3′ tetraethylbenzimidazolcarbocyanine iodide
BSA: Bovine serum albumin
ddH2O: Double-distilled water
OM: Oligomannuronates
IAPP: Human Islet amyloid polypeptide
JNK: c-Jun N-terminal protein kinase
DCFH2-DA: 2,7′-Dichlorofluorescin diacetate
